# Outbreak of Extended-Spectrum Beta-Lactamase Producing *Enterobacter cloacae* with High MICs of Quaternary Ammonium Compounds in a Hematology Ward Associated with Contaminated Sinks

**DOI:** 10.3389/fmicb.2016.01070

**Published:** 2016-07-12

**Authors:** Angélique Chapuis, Lucie Amoureux, Julien Bador, Arthur Gavalas, Eliane Siebor, Marie-Lorraine Chrétien, Denis Caillot, Marion Janin, Claire de Curraize, Catherine Neuwirth

**Affiliations:** ^1^Laboratoire de Bactériologie Médicale et de Surveillance Environnementale, Hôpital Universitaire de DijonDijon, France; ^2^Service d'Hématologie Clinique, Hôpital Universitaire de DijonDijon, France

**Keywords:** ESBL-producing *Enterobacter cloacae*, outbreak, Hematology ward, contaminated sinks, quaternary ammoniums

## Abstract

**Objective:** To investigate an outbreak of extended-spectrum beta-lactamase (ESBL) producing *Enterobacter cloacae* that occurred in the Hematology ward (24-bed unit) of the François Mitterrand University Hospital (Dijon, France) between January 2011 and December 2013. The outbreak involved 43 patients (10 infected and 33 colonized).

**Design:** We performed environmental analysis to detect multiresistant *E. cloacae* for comparison with clinical isolates (genotyping by pulsed-field gel electrophoresis and MLST as well as ESBL-typing) and determined the MICs of the quaternary ammonium compounds (QACs) alkyldimethylbenzylammonium chloride (ADBAC) and didecyldimethylammonium chloride (DDAC). A bleach-based cleaning-disinfection program was implemented in December 2012 after mechanical removal of the biofilm in all sinks.

**Results:** We have detected 17 ESBL-producing *E. cloacae* in patients sink drains, shower drains and medical sink drains. Sequencing of the *bla* genes performed on 60 strains recovered from patients and environment (*n* = 43 clinical and *n* = 17 environmental) revealed that *bla*_CTX−M15_ was predominant (37 isolates) followed by *bla*_CTX−M9_ plus *bla*_SHV−12_ (20 isolates). We observed a great diversity among the isolates: 14 pulsotypes (11 STs) in clinical isolates and 9 pulsotypes (7 STs) in environmental isolates. Six pulsotypes were identical between clinical and environmental isolates. MICs of the quaternary ammonium compounds widely used for disinfection were very high in clinical and environmental isolates. Immediately after the implementation of the disinfection program we noticed a substantial fall in cases number. Our findings demonstrate the role of drains as important reservoir of ESBL-producing *E. cloacae* and highlight the necessity to settle drains accessible to achieve correct cleaning as well as to use disinfectant with proved activity against nosocomial pathogens.

## Introduction

The emergence and spread of multidrug resistance among Gram negatives is a worldwide public-health concern limiting the therapeutic choice to treat severe community and hospital acquired infections. Resistance to broad-spectrum cephalosporins among *Enterobacteriaceae* is mostly related to acquisition of extended-spectrum beta-lactamases (ESBLs) (Pitout and Laupland, 2008). The epidemiology of ESBL-producing strains has evolved: initially ESBLs were usually TEM or SHV derivatives but since the 2000s ESBLs of CTX-M family became predominant (Bonnet, 2004). ESBL-producers are mostly *Escherichia coli* and *Klebsiella pneumoniae* (Tal Jasper et al., 2015) and many outbreaks have been reported (Paterson and Bonomo, 2005). Concerning *Enterobacter cloacae* the frequency of ESBL-producers among isolates that overexpress the chromosomal beta-lactamase is probably underestimated (Paterson and Bonomo, 2005). Anyway there is an increasing report of health care-associated infections due to multiresistant isolates (Mezzatesta et al., 2012). According to Rice et al. *Enterobacter* species belong to the “ESKAPE” pathogens (*Enterococcus faecium, Staphylococcus aureus, Klebsiella pneumoniae, Acinetobacter baumannii, Pseudomonas aeruginosa* and *Enterobacter* species) underlining the importance of this clinical pathogen (Rice, 2008; Boucher et al., 2009). Patients who stay in hospital for a long time are at increased risk of acquiring an *E. cloacae* infection, especially those in intensive care units (ICUs) (Wisplinghoff et al., 2004), in neonatal units (Modi et al., 1987; Mulgrave, 1991; Dalben et al., 2008) and those suffering from hematologic malignancy (Sanders and Sanders, 1997; Bousquet et al., 2014).

*E. cloacae* infections can be acquired from endogenous source (colonization of the gastrointestinal tract) or exogenous sources (Chang et al., 2013). Exogenous factors include the environment of the health care center where the patient is hospitalized (Sanders and Sanders, 1997; Matoušková and Holy, 2014). Nevertheless the particular role of the environment in transmission of ESBL-producing strains to patients has not been widely investigated (Guet-Revillet et al., 2012; Gbaguidi-Haore et al., 2013).

In this study we describe a large outbreak of ESBL-producing *E. cloacae* in a Hematology ward and report the results of the environmental investigations.

## Materials and methods

### Clinical setting

At the time of the outbreak (January 2011-December 2013) the Hematology ward of the teaching hospital of Dijon François Mitterrand, France (1600 beds) included a 15-bed conventional unit and a 9-bed unit under protective isolation. Both units were separated with sliding doors. Each unit had a distinct nursing room, a distinct nurse staff but shared the same medical staff. All patients were treated for hematologic malignancies and prior to intensive chemotherapy a central venous catheter was positioned. Those that underwent prolonged neutropenia or bone marrow transplantation were hospitalized in the protected area.

In the conventional unit (single rooms 3, 4, 32, 34, 36, 37, 38, 40, 41, 42, 44, 46, 48, 64, 65) there was a handbasin at the entrance of the room for staff and visitors. Next to each room there were a shower stall and toilets. In the 9 patient rooms under protective isolation (single rooms 8, 10, 12, 17, 19, 20, 24, 26, 30), air was filtered through a laminar flow system. All members of the staff and all visitors had to wash hands in a devoted handbasin and to wear surgical mask and gown before entering the room. A washbasin and toilets were located next to the bed for patient use.

All taps of the ward were provided with antibacterial filters and for hand washing the staff used mostly soap and water or alcohol-based rinse.

Before the outbreak all surfaces surrounding the patients used to be cleaned with a solution containing quaternary ammonium compounds (QACs): didecyldimethylammonium chloride (DDAC, 0.25%). This disinfectant was also daily poured into all sinks. After hospital environment investigations had been performed, a bleach-based cleaning-disinfection programme was implemented in December 2012. First the biofilm was removed from all drains (handbasins, showers, sinks) and then a bleach solution was poured daily.

### Patient samples

This study was carried out in accordance with the recommendations of the Ethics Committee of the University Hospital François Mitterrand of Dijon. All subjects gave written informed consent in accordance with the Declaration of Helsinki. Swab throat, expectoration, urine and stool cultures were performed weekly. Blood cultures were performed daily (and more in case of fever). We included in the study a single isolate per patient (the first recovered whatever the site).

### Environmental samples

In November 2012 about 100 environmental samples were collected. Ten water samples were collected from different taps (nursing rooms, medication preparation rooms and some patient rooms). The water (100 ml) was filtered and cultured on Drigalski agar. For dry surfaces (beds, doors and tables) we used Count-Tact® agar (bioMérieux, Marcy l'Etoile, France). We also collected 73 samples from drains. For this purpose we introduced a sterile cotton swab to a depth of 5 cm in the drain and performed circular sweeps. The samples were inoculated on Drigalski agar plates supplemented with ceftazidime (4 mg/l). In each room of the protected area we took samples from: handbasin (drain), washbasin (drain), toilets (bowl). We also collected in this unit a sample of the sink drain from the medication room. In the rooms of conventional unit we analyzed drains from handbasin, washbasin and shower.

### Bacterial identification and antibiotic susceptibility testing

Isolates were identified with API20E strips (bioMérieux, Marcy l'Etoile, France).

Antibiogram was performed by the disc diffusion method on Mueller–Hinton agar (bioMérieux) according to the guidelines of the CLSI (Clinical Laboratory Standards Institute, 2012). The production of ESBL was investigated by the double-disc synergy test as already described on Mueller-Hinton agar without and with cloxacillin (250 mg/l) (Jarlier et al., 1988).

### Analytical isoelectric focusing

After overnight culture beta-lactamases were extracted from bacteria by sonication. Unbroken cells and cell envelopes were removed by centrifugation. Isoelectric focusing was performed on polyacrylamide gels containing ampholines with a pH range of 3.5–10. The beta-lactamase activity was located on the gels by an iodine starch procedure after overnight migration (Labia et al., 1976).

### Molecular characterization of the ESBL

We searched for the presence of ESBL belonging to the TEM, SHV, CTX-M, PER, and VEB families according to the results of the pI determination. Polymerase chain reaction (PCR) and sequencing of PCR products were performed as already described (Siebor and Neuwirth, 2011).

### Genotyping analysis by pulsed field gel electrophoresis (PFGE) and MLST

The genotypic analysis of the strains has been performed by PFGE after digestion by *Xba*I restriction endonuclease. Electrophoresis was carried out at 5.4 V/cm during 20 h, with pulse-times ranging from 5 to 50 s using the CHEF-DR® II system (Bio-Rad). The interpretation of the results was performed according to Tenover's criteria (Tenover et al., 1995). The different pulsotypes have been identified with a letter and “Ø” indicated a pulsotype different from all patterns identified to date in our hospital. MLST was carried out and analyzed as previously described (Izdebski et al., 2015).

### Determination of minimum inhibitory concentrations of QACs

Two compunds were chosen for this study: alkyldimethylbenzylammonium chloride (ADBAC) and didecyldimethylammonium chloride (DDAC) kindly provided by Laboratoire Anios® (Hellemmes, France). They are QACs commonly included in products used for decontamination of surface environment in hospital or for hand washing of healthcare workers.

The MICs of ADBAC and DDAC were determined as already described (Buffet-Bataillon et al., 2011) in Mueller Hinton agar containing 0–512 mg/l of QACs. Spots (10^4^ cfu/spot) were distributed with a replicating device (MIC 2000, Dynatech Laboratories Inc., Virginia, USA). Plates were incubated overnight at 37⋅C. The four reference strains (*Staphylococcus aureus* CIP 483, *Escherichia coli* CIP 54127, *E. cloacae* CIP 6085 and *Pseudomonas aeruginosa* CIP 103467) were included to all series. We also studied 17 clinical isolates of *E. cloacae* recovered in different wards of our hospital with various profiles of resistance to beta-lactams (13 wild-type, 2 overexpression of AmpC, 2 ESBL). The MIC was defined as the lowest concentration of QACs for which no bacterial growth was observed. All experiments were performed in triplicate.

## Results

Results are summarized in Table 1 (clinical strains), Table 2 (environmental strains), and Table S1 (Supplementary data).

**Table 1:** **Characteristics of ESBL-producing *Enterobacter cloacae* isolated from patients hospitalized in the Hematology ward**.

**Patient number**	**Strain**	**Date of first isolation (dd/mm/yyyy)**	**Source of first isolation**	**ESBL**	**PFGE pulsotype^a^**	**ST**	**Active antibiotics^b^**	**ADBAC MIC (mg/l)**	**DDAC MIC (mg/l)**
P1	EcP1	03/02/2011	Stool	CTX-M-9/SHV-12	B	133	IPM, DOR, MEM, AMK, TGC, FOF	ND	ND
P2	EcP2	21/02/2011	Stool	CTX-M-15	C	114	IPM, DOR, MEM, AMK, FOF	ND	ND
P3	EcP3	07/03/2011	Stool	CTX-M-15	C	114	IPM, DOR, MEM, TGC, FOF	ND	ND
P4	EcP4	18/05/2011	Blood	CTX-M-15	C	114	IPM, DOR, MEM, AMK, FOF	ND	ND
P5	EcP5	10/10/2011	Stool	CTX-M-9/SHV-12	Ø	50	IPM, DOR, MEM, AMK, FOF	ND	ND
P6	EcP6	17/10/2011	Stool	CTX-M-15	C	114	IPM, DOR, MEM, AMK, FOF	ND	ND
P7	EcP7	19/01/2012	Stool	CTX-M-9/SHV-12	D	50	IPM, DOR, MEM, AMK	ND	ND
P8	EcP8	13/02/2012	Stool	CTX-M-9/SHV-12	E	110	IPM, DOR, MEM, FOF	256	128
P9	EcP9	27/02/2012	Throat	CTX-M-9/SHV-12	D	50	IPM, DOR, MEM, AMK, FOF	128	64
P10	EcP10	26/03/2012	Blood	CTX-M-9/SHV-12	A	110	IPM, DOR, MEM, AMK, OFX, CIP, TGC, FOF	256	128
P11	EcP11	01/05/2012	Blood	CTX-M-9/SHV-12	D	50	IPM, DOR, MEM, AMK, FOF	>512	512
P12	EcP12	31/05/2012	Stool	CTX-M-9/SHV-12	B	133	IPM, DOR, MEM, FOF	256	128
P13	EcP13	11/06/2012	Stool	CTX-M-15	F	66	IPM, DOR, MEM, AMK, FOF	128	128
P14	EcP14	18/06/2012	Stool	CTX-M-15	C	114	IPM, DOR, MEM, AMK, FOF	256	128
P15	EcP15	05/07/2012	Throat	CTX-M-9/SHV-12	B	133	IPM, DOR, MEM, CIP, TGC, FOF	256	128
P16	EcP16	09/08/2012	Blood	CTX-M-15	C	114	IPM, DOR, MEM, AMK, FOF	64	64
P17	EcP17	20/08/2012	Stool	CTX-M-15	C	114	IPM, DOR, MEM, AMK, TGC, FOF	256	128
P18	EcP18	23/08/2012	Throat	CTX-M-9/SHV-12	Ø	50	IPM, DOR, MEM, CIP	128	64
P19	EcP19	30/08/2012	Stool	CTX-M-9/SHV-12	D	50	IPM, DOR, MEM, AMK, FOF	512	512
P20	EcP20	26/09/2012	Thoracic wound	CTX-M-15	C	114	IPM, DOR, MEM, AMK, FOF	256	128
P21	EcP21	29/10/2012	Urines	CTX-M-15	C	114	IPM, DOR, MEM, FOF	256	128
P22	EcP22	08/11/2012	Stool	CTX-M-15	F	66	IPM, DOR, MEM, FOF	256	128
P23	EcP23	12/11/2012	Stool	CTX-M-15	Ø	45	IPM, DOR, MEM, AMK, OFX, CIP, FOF	256	128
P24	EcP25	22/11/2012	Urines	CTX-M-15	F	66	IPM, DOR, MEM, AMK, FOF	256	128
P25	EcP26	26/11/2012	Stool	CTX-M-15	F	66	IPM, DOR, MEM, AMK, FOF	256	128
P26	EcP27	28/11/2012	Blood	CTX-M-15	F	66	IPM, DOR, MEM, AMK, FOF	128	128
P27	EcP28	06/12/2012	Throat	CTX-M-15	F	66	IPM, DOR, MEM, AMK, FOF	256	128
P28	EcP29	13/12/2012	Throat	CTX-M-9/SHV-12	D	50	IPM, DOR, MEM, AMK, CIP, FOF	128	64
P29	EcP30	13/12/2012	Throat	CTX-M-15	F	66	IPM, DOR, MEM, AMK, FOF	256	128
P30	EcP31	17/12/2012	Stool	CTX-M-15	C	114	IPM, DOR, MEM, TGC, FOF	128	128
P31	EcP32	20/12/2012	Urines	CTX-M-15	Ø	97	IPM, DOR, MEM, AMK, FOF	256	512
P32	EcP33	18/02/2013	Stool	CTX-M-15	Ø	78	IPM, DOR, MEM, AMK, FOF	>512	>512
P33	EcP34	24/03/2013	Stool	ND	Ø	NEW	IPM, DOR, MEM, KAN, TOB, AMK, GEN, NET, CIP, FOF	ND	ND
P34	EcP35	10/05/2013	Stool	CTX-M-9/SHV-12	B	133	IPM, DOR, MEM, FOF	ND	ND
P35	EcP36	13/06/2013	Stool	CTX-M-15	C	114	IPM, DOR, MEM, AMK, TGC, FOF	ND	ND
P36	EcP37	24/06/2013	Urines	SHV-12	Ø	106	IPM, DOR, MEM, AMK, FOF	ND	ND
P37	EcP38	01/07/2013	Throat	CTX-M-15	C	114	IPM, DOR, MEM, AMK, TGC, FOF	ND	ND
P38	EcP39	15/07/2013	Throat	CTX-M-15	C	114	IPM, DOR, MEM, AMK, FOF	ND	ND
P39	EcP40	08/08/2013	Stool	CTX-M-9/SHV-12	D	50	IPM, DOR, MEM, AMK, OFX, CIP, TGC, FOF	ND	ND
P40	EcP41	23/09/2013	Stool	CTX-M-15	C	114	IPM, DOR, MEM, TGC, FOF	ND	ND
P41	EcP42	17/10/2013	Stool	ND	Ø	145	IPM, DOR, MEM, AMK, GEN, NET, OFX, CIP, TGC, SXT, FOF	ND	ND
P42	EcP43	16/12/2013	Stool	CTX-M-15	F	66	IPM, DOR, MEM, AMK, FOF	ND	ND
P43	EcP44	12/12/2013	Stool	CTX-M-15	F	66	IPM, DOR, MEM	ND	ND

a *Letters identify the different pulsotypes. A pulsotype that was recovered only once is reported by “Ø”*.

b *AMK, amikacin; CIP, ciprofloxacin; FOF, fosfomycin; GEN, gentamicin; IPM, imipenem; KAN, kanamycin; NET, netilmicin; OFX, ofloxacin; TGC, tigecycline; TOB, tobramycin; SXT, trimethoprim-sulfamethoxazole*.

*ND, not determined*.

**Table 2:** **Characteristics of ESBL-producing *Enterobacter cloacae* isolated from the environment of patients hospitalized in the Hematology ward**.

**Source**	**Sample**	**Strain**	**PFGE pulsotype^a^**	**ST**	**ESBL**	**ADBAC MIC (mg/l)**	**DDAC MIC (mg/l)**
**PROTECTED AREA**							
Room 19	Patient sink drain	EcE1	**F**	66	CTX-M-15	128	128
**CONVENTIONAL AREA**							
Room 34	Medical sink drain	EcE2	**C**	114	CTX-M-15	ND	ND
	Patient sink drain	EcE3	**C**	114	CTX-M-15	ND	ND
	Shower drain	EcE4	**C**	114	CTX-M-15	256	128
		EcE5	**C**^b^	114	CTX-M-15	128	128
Room 36	Medical sink drain	EcE6	**B**	133	CTX-M-9/SHV-12	256	128
	Shower drain	EcE7	**A**	110	CTX-M-9/SHV-12	512	512
Room 37	Shower drain	EcE8	**D**	50	CTX-M-9/SHV-12	512	512
Room 38	Patient sink drain	EcE9	**F**	66	CTX-M-15	128	128
	Shower drain	EcE10	**Ø**	50	CTX-M-9/CTX-M-15/SHV-12	128	64
Room 40	Shower drain	EcE11	**E**	110	CTX-M-9/SHV-12	128	128
Room 46	Shower drain	EcE12	**F**	66	CTX-M-15	128	64
		EcE13	**C**	114	CTX-M-15	128	128
Room 48	Shower drain	EcE14	**E**	110	CTX-M-9/SHV-12	256	256
Room 64	Shower drain	EcE15	**C**	114	CTX-M-15	128	128
Room 65	Shower drain	EcE16	**Ø**	78	CTX-M-15	128	64
		EcE17	**Ø**	NEW	CTX-M-9/SHV-12	256	128

a *Letters identify the different pulsotypes. A pulsotype that was recovered only once is reported by “Ø”. Pulsotypes in bold were identical to clinical strain pulsotypes*.

b *Isolate resistant to carbapenems*.

*ND, not determined*.

### Outbreak

**Figure 1:**
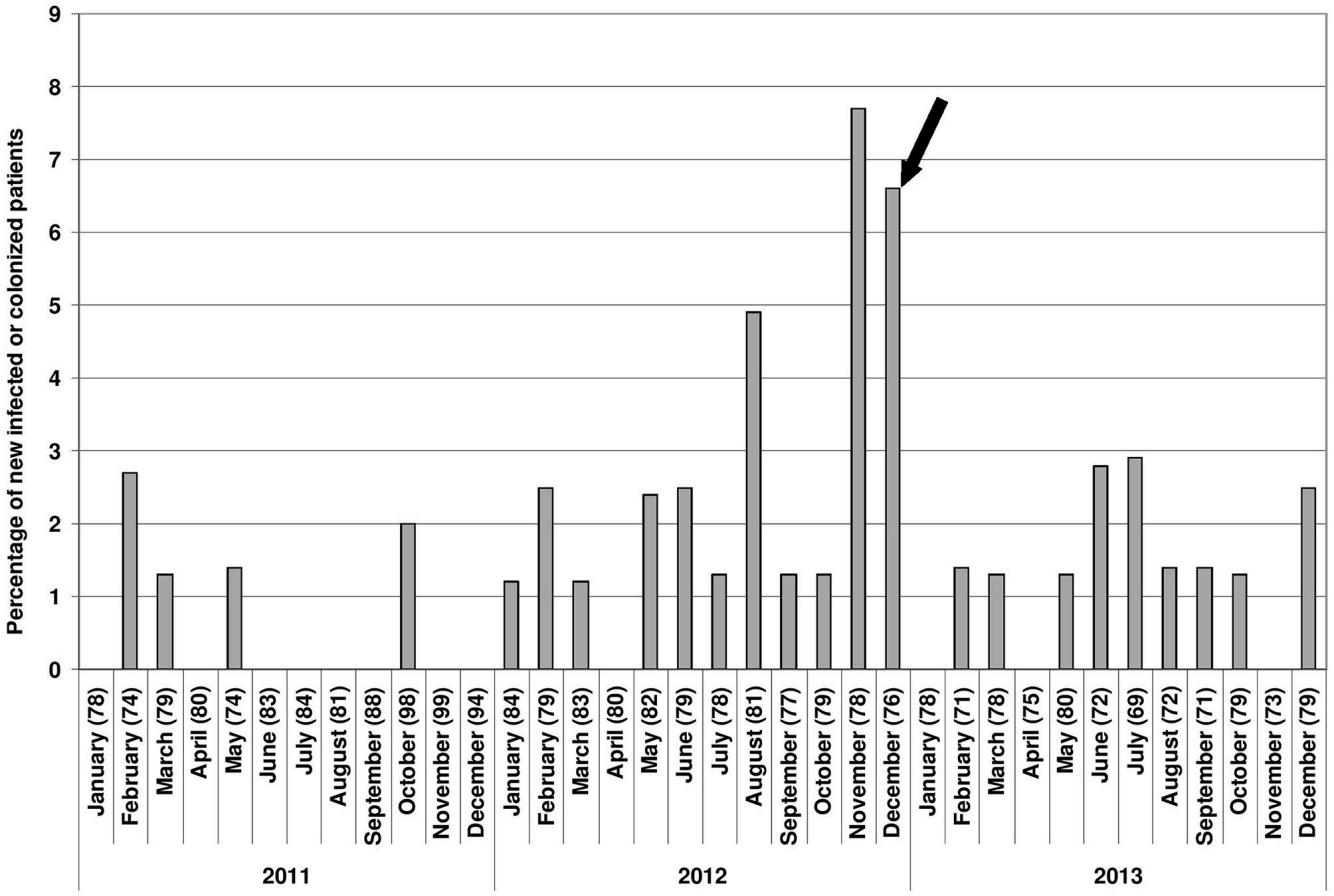
**Epicurve with all samples of *E. cloacae* for newly infected or colonized patients between January 2011 and December 2013**. Numbers in parenthesis are the numbers of newly hospitalized patients in the Hematology ward each month. Black arrow: implementation of the bleach-based disinfection programme after complete cleaning of the sink.

A total of 43 ESBL-producing *E. cloacae* have been isolated from 43 patients hospitalized in the Hematology ward between January 2011 and December 2013: 6 isolates in 2011, 25 in 2012 and 12 in 2013 (Figure 1). Most of the isolates have been recovered from stool but also in other sites such as throat, urine, blood (respectively 25, 8, 4, and 5) and in 1 thoracic wound (infection of the insertion site of the central catheter). The 10 patients by whom the ESBL-producing *E. cloacae* had been recovered from urine, thoracic wound and blood harbored clinical symptoms of infection. All other patients were considered as colonized.

### Environmental isolates

Seventeen ESBL-producing *E. cloacae* have been detected in environmental samples. Most of them (16) have been recovered in the conventional unit and only one in the protected area. In the conventional unit the resistant organism has been found in 9 patients rooms. All isolates have been detected in wet environment: sink drains (medical sink drain and patient sink drain) as well as in many shower drains.

### Genotyping of the isolates

The genotypic diversity of *E. cloacae* isolates is shown in Tables 1, 2. The 43 strains isolated from patients belonged to 14 pulsotypes (Figure S1) and 11 STs. Pulsotype C was the most frequently encountered (14 strain). More than half of the isolates belonged to ST114 or ST66. Since 2012 we observed a diversification of the isolates with an increase in the number of pulsotypes and the emergence of pulsotypes D and F. Isolates that belonged to pulsotype C, D, or F represented 70% of all clinical isolates over the 3 years.

Among the 17 environmental ESBL-producing *E. cloacae* there were 9 distinct pulsotypes and 7 STs. Among the 9 pulsotypes, 6 were identical to those of patients isolates (pulsotypes A, B, C, D, E, and F) with the same predominance of pulsotypes C and F. Generally, in the rooms of conventional unit several samples were positive with the exception of the rooms 37, 48, 64, and 65. In the room 34 the three samples were positive, indicating a wide contamination.

Several rooms were contaminated by strains belonging to different pulsotypes (rooms 36, 38, 46, and 65). In some samples we identified strains belonging to different pulsotypes such as in the shower drains of the rooms 46 and 65.

### ESBL characterization

Sequencing of the *bla* genes has been performed on 58 ESBL-producing *E. cloacae* isolated from patients and environment. All isolates belonging to the same genotype harbored the same ESBL. Thirty-seven isolates belonging to pulsotype C, F, or Ø (respectively 21, 12, and 4 strains) harbored CTX-M-15 whereas 20 isolates (belonging to various pulsotypes) harbored both CTX-M-9 and SHV-12. One isolate (EcE10) recovered in a shower drain produced three ESBL: SHV-12, CTX-M-9, and CTX-M-15 and was genetically unrelated.

### Susceptibility testing

All clinical isolates were resistant to all beta-lactams with the exception of carbapenems. Amikacin remained active in most cases (73%) as well as fosfomycin (89%). Only 25% of the isolates were susceptible to tigecyclin and 20% to ciprofloxacin. The MICs of ADBAC and DDAC were very high for clinical and environmental isolates, ranging from 64 to > 512 mg/l. The MICs of ADBAC and DDAC for the four reference strains were respectively 2 and 1 mg/l for *S. aureus* CIP 483 32, and 8 mg/l for *E. coli* CIP 54127, 64 and 64 mg/l for *E. cloacae* CIP 6085, 512 and 512 mg/l for *P. aeruginosa* CIP 103467.

## Discussion

Our investigations started when we observed that the number of patients with ESBL-producing *E. cloacae* increased dramatically since August 2012. Indeed 17 new patients were concerned between August and December 2012.

It is noteworthy that many isolates were identified as ST114 or as ST66. In a very recent study these STs that belong to the same apparent clonal complex CC114 (Izdebski et al., 2015) have been described as prevalent and widespread. Indeed they have been detected from rectal swabs in several hospital units across Europe (France, Italy, Spain, Greece) but also Israel. The biological success of these clones has to be confirmed by undertaking MLST analysis of a large number of isolates from different areas.

The genotypic diversity of our clinical isolates did not suggest a single common exogenous source of infection as already noticed in previous studies about outbreaks of *P. aeruginosa* linked with environmental contamination (Inglis et al., 2010). Moreover, the observation of the practices of health-care workers revealed that they complied with the infection control measures. This prompted us to conduct investigation in the patient care environment. Surprisingly ESBL-producing *E. cloacae* have been detected in 10 patients rooms, meaning an environmental contamination of nearly half of the total ward. Positive samples concerned only the sink drains (medical sink drains, patient sink drains, and shower drains). In these sites we observed the presence of biofilm. We did not detect any ESBL-producing *E. cloacae* on wet surfaces or in the water. That is not in accord with other reports (Gbaguidi-Haore et al., 2013; Judge et al., 2013). For instance in the study of Judge et al., the culture of 4 out of 18 wet sites surrounding patients colonized by ESBL-producing *K. pneumoniae* were positive with the same organism (Judge et al., 2013). Most of the descriptions of outbreaks due to ESBL-producing *E. cloacae* report characterization of the clinical isolates but environmental sampling of wet sites around the patient are rarely performed (Dalben et al., 2008; Kruse et al., 2010). Nevertheless, two interesting studies compared environmental and clinical isolates belonging mostly to other species. In the first one Lowe et al. reported an outbreak of ESBL-producing *Klebsiella oxytoca* concerning 66 patients and demonstrated that the sink drains were reservoirs. The outbreak stopped after sink drain modification (Lowe et al., 2012). In the second one comparing 62 environmental strains belonging to 4 species of ESBL-producing *Enterobacteriaceae* with 43 clinical isolates, the authors identified 4 identical patterns (3 *K. oxytoca* and 1 *E. cloacae*) between clinical and environmental strains recovered from wet sites including sink drains (Kac et al., 2004). Our findings confirm that sink drains have to be considered as important potential reservoirs of ESBL-producing strains. This has been also pointed out in the study of Roux et al. that reported the contamination by ESBL-producing *Enterobacteriaceae* of 57 sinks among the 185 that were sampled (Roux et al., 2013). Interestingly these producers were mainly *K. pneumoniae* but also *E. cloacae* (respectively 33 and 18). Unfortunately the study did not report comparison of these environmental strains with clinical isolates.

**Figure 2:**
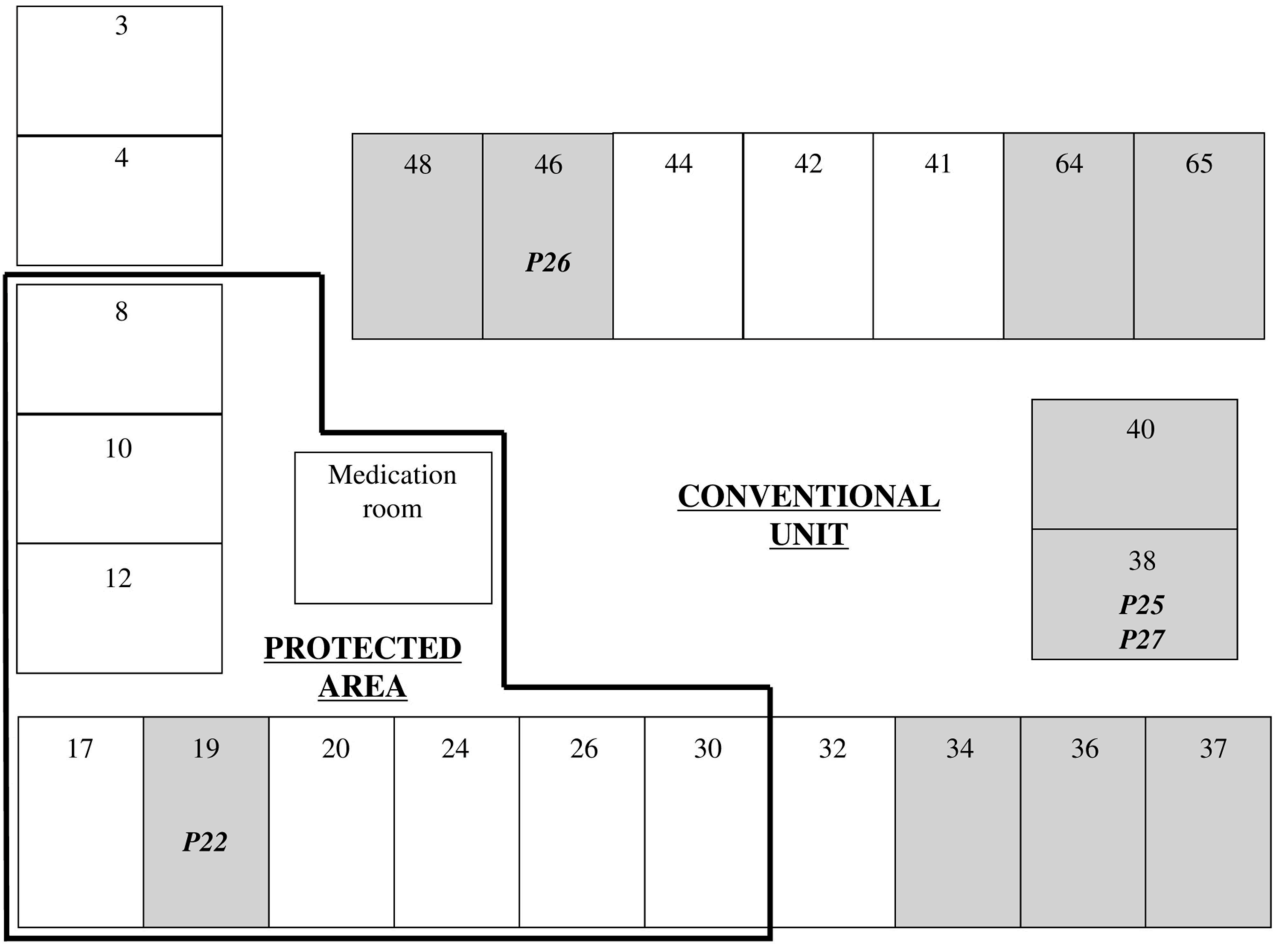
**Plan of the Hematology ward**. Numbers indicate room numbers. ESBL-producing *E. cloacae* were isolated from samples collected in gray rooms. Patient number contaminated by sink is indicated in the occurring room.

The transfer of pathogens from sink to patients may occur in several situations resulting from a splash-back that leads to contamination of an area that can reach at least 1 m from the sink (Hota et al., 2009). Therefore, the patient can be contaminated during teeth brushing or hands washing or showering. The health-care workers are also at risk of hand contamination leading possibly to transfer the pathogen to the patients during care. The phenomenon is amplified when the water flows directly into the sink drain as we observed in nearly all patients rooms. This is considered as inadequate sink design (Breathnach et al., 2012). The mode of contamination is difficult to establish for most of our patients mainly because of hospital stays in different rooms. Nevertheless, for 4 of them the correlation between their colonization/infection and their stay in a contaminated room was easy to perform (Figure 2).

The first one (P22) was in room 32 from 16th to 31st October 2012. All samples from stool and throat were negative (each performed 4 times). He has been transferred in the protected area in the room 19 (1st of November) and had twice negative samples. An ESBL-producing *E. cloacae* has been detected in his stool on the 8th of November. This isolate belonged to pulsotype F as the one from the sink drain of room 19 suggesting that the contamination occurred there. The second one (P25) had 4 hospital stays between the 21st June 2012 and the 29th September 2012 for a total of 69 days. During this period he had 17 negative samples. Between the 16th October and the 30th November he was successively in rooms 38, 64, and 30. On the 26th November 2012 he had a positive stool sample that harbored the pulsotype F as the one from the sink drain of room 38. The third one (P27) had 7 hospital stays between the 2nd April 2012 and the 3rd December 2012 in different rooms with 23 negative samples. He was transferred in room 38 on the 4th December and we detected an ESBL-producing *E. cloacae* in his throat only 2 days later. In this case the contamination occurred probably while brushing his teeth. For the fourth patient (P26) the series of events is easier. He arrived at hospital on the 21st November 2012. The same day a PICC-line catheter was positioned. He stayed in room 46 and had twice negative samples (22nd and 26th November). On the 28th November he developed a sepsis and blood cultures were positive with ESBL-producing *E. cloacae* that belonged to pulsotype F as the one recovered in the shower drain in room 46. The contamination of this patient probably occurred while he took a shower. At this occasion splash-back from the drain might have transfer the pathogen to the dressing of the catheter inducing then a sepsis for which he had to be transferred in ICU.

All these findings and the fact that substantial fall in cases number has been observed after complete cleaning of the sinks in the ward indicated that they were very important reservoirs. Nevertheless, a last question rose: why *E. cloacae* isolates belonging to various pulsotypes were so widespread? It is well established that organisms are able to survive in biofilm (Khan et al., 2012; Vergara-López et al., 2013). MICs of ADBAC and DDAC were very high for all isolates of *E. cloacae* included whatever their beta-lactam resistance phenotype: ESBL, overexpression of AmpC, wild-type but also the reference strain. These very first data about the efficacy of the QACs on *E. cloacae* suggested that this species might be intrinsically poorly susceptible to these compounds. The genetic determinants of this resistance remain to be explored, especially the presence of efflux pump genes as already described for *E. coli* and *K. pneumoniae* (Abuzaid et al., 2012; Buffet-Bataillon et al., 2012).

In the Hematology ward the disinfectant poured daily into all sinks was at the recommended concentration of 0.25% (or 2500 mg/l). Given that the sinks were covered with biofilm and that the time of contact of that biofilm to the disinfectant was short the organisms present in the sinks were exposed to subinhibitory concentrations and survived despite the frequent disinfection. In a previous study it was demonstrated that QAC resistance could be enhanced through exposure of the sensitive bacteria to increasing concentrations of QACs (Sidhu et al., 2002). The disinfection program implemented in December 2012 included biofilm removal from all sinks and use of a bleach solution daily. These measures were efficient: only three new cases occurred between January and May 2013. After June 2013 we observed a new minor increase of the number of cases. The sinks are not accessible without complete dismantling and therefore their complete cleaning was not performed again, leading to the formation of new biofilm and to the risk of persistence of dangerous reservoirs. The susceptibility to disinfectants of diverse multiresistant nosocomial pathogens might be tested by the manufacturers to provide the best information to the users.

In conclusion, in this study we have demonstrated that the large outbreak of ESBL-producing *E. cloacae* concerning 43 patients from the Hematology ward was associated with the contamination of many sinks of the unit by isolates with decreased susceptibility to QACs. It is necessary to keep in mind that all components of hospital environment are important in the prevention of healthcare-associated infections and this includes the choice of accessible sinks to allow a periodic cleaning with efficient disinfectant. “*Primum non nocere*” is a guiding principle for physicians but should be also a guiding principle for hospital designers.

## Author contributions

AC performed isolation of resistant organisms and sequencing of bla genes. LA, ES performed PFGE. JB, MJ determined quaternary ammonium MICs. CD performed environmental investigation. MC, DC provided clinical data. CN designed the study, chose the methods and wrote the manuscript. AG performed MLST.

### Conflict of interest statement

The authors declare that the research was conducted in the absence of any commercial or financial relationships that could be construed as a potential conflict of interest.

## Supplementary material

Figure S1 **Illustration of the diversity of the PFGE profiles among *E. cloacae* isolates**.

Table S1 **MICs of QACs for clinical *E. cloacae* strains recovered in our hospital**. ^a^AmpC++, overexpressed AmpC; ESBL, extended-spectrum β-lactamase; WT, wild type.
