# Supplementary figures and images for: Outbreak of Extended-Spectrum Beta-Lactamase Producing Enterobacter cloacae with High MICs of Quaternary Ammonium Compounds in a Hematology Ward Associated with Contaminated Sinks
Source: Front Microbiol. 2016 Jul 12;7:1070. doi: 10.3389/fmicb.2016.01070 (PMC4940370; doi:10.3389/fmicb.2016.01070)

Figure 3

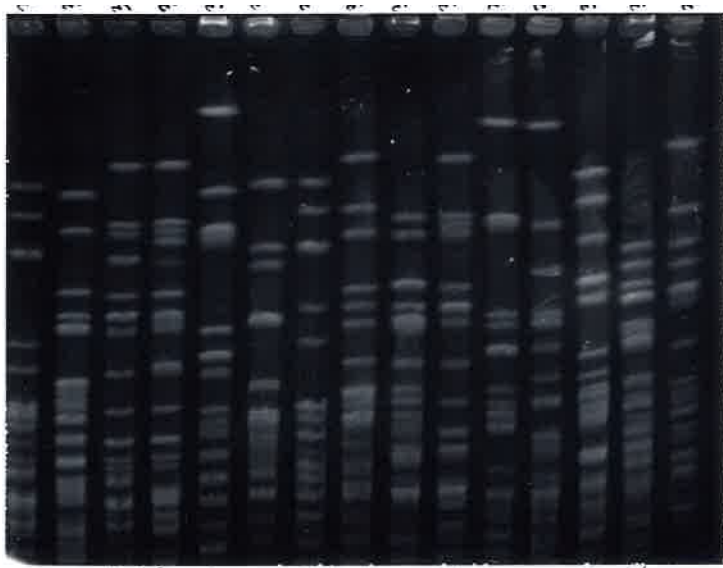

Supplement: Figure S1 — Illustration of the diversity of the PFGE profiles among E. cloacae isolates. [file Image1.PDF]
