# Supplementary material for: Outbreak of Extended-Spectrum Beta-Lactamase Producing Enterobacter cloacae with High MICs of Quaternary Ammonium Compounds in a Hematology Ward Associated with Contaminated Sinks
Source: Front Microbiol. 2016 Jul 12;7:1070. doi: 10.3389/fmicb.2016.01070 (PMC4940370; doi:10.3389/fmicb.2016.01070)
Supplement: Table S1 — MICs of QACs for clinical E. cloacae strains recovered in our hospital. aAmpC++, overexpressed AmpC; ESBL, extended-spectrum β-lactamase; WT, wild type. [file Table1.doc]

**Table 3**

| Strain | Ward source | Date of isolation (dd/mm/yyyy) | Source of isolation | β-lactams resistance phenotype^a^ | ADBAC MIC (mg/l) | DDAC MIC (mg/l) |
| --- | --- | --- | --- | --- | --- | --- |
| EcP45 | Pneumology | 18/09/2012 | Pleural fluid | WT | 64 | 64 |
| EcP46 | Medecine | 07/12/2012 | Wound | AmpC++ | 256 | 64 |
| EcP47 | Hematology | 13/12/2012 | Stool | WT | 256 | 64 |
| EcP48 | Hematology | 13/12/2012 | Throat | WT | 128 | 64 |
| EcP49 | Hematology | 27/12/2012 | Skin biopsy | WT | 256 | 64 |
| EcP50 | Hematology | 27/12/2012 | Stool | WT | 64 | 64 |
| EcP51 | Hematology | 31/12/2012 | Throat | WT | 256 | 128 |
| EcP52 | ICU-1 | 06/01/2013 | Sputum | WT | 256 | 128 |
| EcP53 | Pediatrics | 05/02/2013 | Wound | WT | 256 | 128 |
| EcP54 | ICU-2 | 06/02/2013 | Sputum | WT | 256 | 64 |
| EcP55 | Hematology | 07/02/2013 | Throat | WT | 128 | 128 |
| EcP56 | Pneumology | 07/02/2013 | Stool | ESBL | 256 | 128 |
| EcP57 | Cardiology | 08/02/2013 | Wound | WT | 256 | 64 |
| EcP58 | Emergency | 09/02/2013 | Wound | WT | 256 | 64 |
| EcP59 | ICU-3 | 13/02/2013 | Catheter | ESBL | 128 | 128 |
| EcP60 | Pediatrics | 14/02/2013 | Stool | WT | 256 | 64 |
| EcP61 | Medecine | 20/02/2013 | Catheter | AmpC++ | 64 | 64 |

Table 3

MICs of QACs for clinical *E. cloacae* strains recovered in our hospital

^a^ AmpC++: overexpressed AmpC, ESBL : extended-spectrum β-lactamase, WT: wild type
